# METTL3-induced circ_0008345 contributes to the progression of colorectal cancer via the microRNA-182-5p/CYP1A2 pathway

**DOI:** 10.1186/s12885-024-12474-5

**Published:** 2024-06-14

**Authors:** Chaofeng Hou, Jinbo Liu, Junwei Liu, Danjie Yao, Fang Liang, Congpeng Qin, Zhiyong Ma

**Affiliations:** 1 Department of Anorectal Surgery, Zhengzhou Central Hospital Affiliated to Zhengzhou University, No. 195, Tongbai North Road, Zhongyuan District, Zhengzhou, Henan 450000 P.R. China; 2 Department of Colorectal and Anal Surgery, The First Affiliated Hospital of Zhengzhou University, Zhengzhou, Henan 450052 P.R. China; 3 Department of Oncology Rehabilitation, Zhengzhou Central Hospital Affiliated to Zhengzhou University, Zhengzhou, Henan 450000 P.R. China

**Keywords:** Colorectal cancer, Circ_0008345, microRNA-182-5p, CYP1A2, METTL3

## Abstract

**Background:**

Circular RNA (circRNAs) have been found to play major roles in the progression of colorectal cancer (CRC). However, the functions of circ_0008345 (transcribed by PTK2) in regulating CRC development remain undefined. In this study, we aimed to explore the roles and underlying mechanisms of circ_0008345 in CRC.

**Methods:**

RNase R-treated total cellular RNA was used to verify the circular structure of circ_0008345, and a subcellular fractionation assay was performed to detect the subcellular localization of circ_0008345. RNA pull-down and dual-luciferase assays were used to verify the binding relation between microRNA (miR)-182-5p and circ_0008345 and/or CYP1A2. Colony formation assay, EdU, and Transwell assays were performed to detect the biological behavior of CRC cells in vitro, and CRC cells were injected into mice to observe the tumor formation. m6A immunoprecipitation was used to detect the m6A modification of circ_0008345 in CRC cells.

**Results:**

Circ_0008345, upregulated in CRC tissues and cells, was mainly present in the cytoplasm. Circ_0008345 bound to miR-182-5p, and miR-182-5p targeted CYP1A2, an oncogene in CRC. The colony formation, mobility, EdU-positive cell rate in vitro, and tumor growth in mice were inhibited after the knockdown of circ_0008345. However, the suppressing effects of sh-circ_0008345 on CRC and CYP1A2 expression were significantly reversed after further knockdown of miR-182-5p. METTL3 was the m6A modifier mediating circ_0008345 expression, and the suppression of METTL3 reduced the expression of circ_0008345.

**Conclusions:**

METTL3-dependent m6A methylation upregulated circ_0008345, which blocked the inhibitory effect of miR-182-5p on CYP1A2, thereby exacerbating the malignant phenotype of CRC cells.

**Supplementary Information:**

The online version contains supplementary material available at 10.1186/s12885-024-12474-5.

## Background

Globally, colorectal cancer (CRC) represents the third most commonly diagnosed malignancy and the second leading cause of cancer-associated death [1]. Although few molecular biomarkers that can detect CRC at the early stage and also the progression of the disease have been identified, there is a huge gap that needs to be filled to improve the screening, prevention, and management of CRC [2]. Epigenetics, defined as heritable changes in gene expression that do not contribute to permanent alterations in the DNA sequence, has an indispensable role in the pathogenesis of CRC [3]. Noncoding RNAs, which can be categorized into: small ncRNAs (< 200 bp), including microRNA (miRNAs/miRs) and long ncRNAs (lncRNAs, > 200 bp), have a role in the epigenetic regulation in CRC [4, 5]. Circular RNAs (circRNAs) are a group of single-stranded non-coding RNA that is highly abundant in mammalian cells, and the majority of circRNAs are endogenously formed through back-splicing [6]. Compared with linear RNAs, circRNAs have covalently closed circular structures without a 5’ cap structure and a 3’ poly A tail and are much more stable than linear RNAs, which have a high degree of stability and a great potential effect on gene regulation [7]. CircRNAs have the function as miRNA sponges, limiting miRNA action in the transcriptional and posttranscriptional control of gene expression [8]. Therefore, they might serve as specific targets for diagnostic and prognostic detection of CRC [9, 10].

Protein-tyrosine kinase 2 (PTK2; also known as FAK) was discovered three decades ago and has been recognized as a key player in the governance of cell-matrix adhesion and mesenchymal cell migration [11]. Recently, the involvement of PTK2-derived circRNAs has been substantiated in diverse cancers, including CRC, by sponging miRNAs [12–14]. In the present study, we identified a rarely reported circRNA derived from PTK2, circ_0008345 using the bioinformatics prediction tool circbank [15]. Further *in silico* analyses showed that miR-182-5p is a possible downstream biomolecule of circ_0008345 and cytochrome P450 1A2 (CYP1A2) as a target of miR-182-5p. MiR-182-5p was found markedly reduced in CRC tissues and cells, and closely correlated with pathological stage, differentiation, and lymphatic metastasis [16]. CYP1A2 is one of the most important cytochrome enzymes in the liver, and alternative splicing, RNA stability, regulatory miRNAs, and DNA methylation are known to affect the expression of CYP1A2 [17]. The interaction between miR-182-5p and CYP1A2 and the upstream mechanism for circ_0008345 upregulation in the pathogenesis of CRC is still unknown. Therefore, in this study, we detected the effect of circ_0008345 on CRC cell proliferation, migration, and invasion, then characterized the upstream and downstream mechanism of circ_0008345 in CRC.

## Methods

### Human samples

A total of 45 CRC patients were recruited from Zhengzhou Central Hospital Affiliated to Zhengzhou University from August 2021 to May 2022, including 26 males and 19 females. CRC tissues and matched adjacent normal tissue were stored at -80 °C. All patients signed a written informed consent and were diagnosed for the first time. They had completed clinical information and did not suffer from other malignancies. This study was approved by the Medical Ethics Committee of Zhengzhou Central Hospital Affiliated to Zhengzhou University.

### Cultivation and treatment of cells

Human CRC cell lines DLD1 (YS100C) and SW480 (YS284C), and fetal human colon (FHC, YS1779C) were acquired from YaJi Biological (Shanghai, China). Cells were cultured in RPMI 1640 (11,875,119, Gibco, Carlsbad, CA, USA) or DMEM (11,965,092, Gibco) plus 10% FBS, 100 U/mL penicillin, and 100 mg/mL streptomycin at 37 °C in humidified air with 5% CO_2_. Lentiviral vectors harboring short hairpin RNA (shRNA) against circ_0008345 (sh-circ_0008345), METTL3 (sh-METTL3), CYP1A2 (sh-CYP1A2) and their scramble control (sh-NC), miR-182-5p inhibitor and negative control (inhibitor-NC), miR-182-5p mimic and its control (miR-control), circ_0008345 overexpression vector (OE-circ_0008345) and control vector (OE-NC) were all designed and purchased from VectorBuilder (Guangzhou, Guangdong, China). After viral infection or transfection of plasmids, the cells were treated with puromycin (3 μg/μL) for 1 week, and surviving cells were selected as stable cells.

### RNA extraction, reverse transcription, and RT–qPCR

TRIzol reagent (15,596,026, Gibco) was applied to extract RNA from tissues and cells, and PrimeScript RT kit (RR014B, Takara Holdings Inc., Kyoto, Japan) was used for the reverse transcription of cDNAs other than miR-182. The reverse transcription of miR-182-5p or miR-151-3p was performed using TaqMan™ MicroRNA Reverse Transcription Kit (4,366,596, Gibco). Finally, cDNA and specific primers (Table 1) were mixed with ChamQ Universal SYBR qPCR Master Mix (Q711-02, Nanjing Vazyme Biotech Co., Ltd., Nanjing, China), and PCR reactions were performed in the PCR system. The expression of mRNAs and circ_0008345 or the miR-182 was calculated relative to the expression of GAPDH or U6 small nuclear RNA, respectively. The comparative cycle threshold values (2^−ΔΔCt^) were calculated to analyze the expression.

**Table 1:** The oligonucleotides used in this study

Name	Primers for PCR (5’-3’)
β-actin	5’-CACCATTGGCAATGAGCGGTTC-3’
	5’-AGGTCTTTGCGGATGTCCACGT-3’
PTK2	5’-GCCTTATGACGAAATGCTGGGC-3’
	5’-CCTGTCTTCTGGACTCCATCCT-3’
circ_0008345	5’-TGTGCTCTTGGTTCAAGCTG-3’
	5’-TCCTTTTGGCAAATAACGAAT-3’
miR-182-5p	5’-GGCAATGGTAGAACTCAC-3’
	5’-GAACATGTCTGCGTATCTC-3’
miR-151-3p	5’-TCGAGGAGCTCACAGTC-3’
	5’-GAACATGTCTGCGTATCTC-3’
CYP1A2	5’-TCATCCTGGAGACCTTCCGACA-3’
	5’-GCCACTGGTTTACGAAGACACAG-3’
METTL3	5’-CTATCTCCTGGCACTCGCAAGA-3’
	5’-GCTTGAACCGTGCAACCACATC-3’
U6	5’-CTCGCTTCGGCAGCACAT-3’
	5’-TTTGCGTGTCATCCTTGCG-3’

Note: PTK2: protein-tyrosine kinase 2; CYP1A2: cytochrome P450 1A2; METTL3: Methyltransferase-like 3

### Actinomycin D assay

The DLD1 or SW480 cells were treated with 2 mg/L actinomycin D (A4262, Sigma-Aldrich Chemical Company, St Louis, MO, USA) and incubated at 37 °C. The treated cells were collected at 15 min, and RNA was extracted to remove the genomic DNA. The cDNA was synthesized using reverse transcription, and qPCR was performed to detect the level of circ_0008345. Finally, the data were analyzed, and the half-life of the target RNA was calculated.

### Luciferase reporter assay

Based on the binding site between miR-182-5p and circ_0008345 or CYP1A2 3’UTR, wild-type (WT) and mutant (MUT) fragments of circ_0008345 or CYP1A2 3’UTR were ligated to the pmirGLO reporter vector (E1330, Promega) to construct circ_0008345-WT/MUT or CYP1A2 3’UTR-WT/MUT vectors. The reporter vector (circ_0008345-WT/MUT or CYP1A2 3’UTR-WT/MUT) and miR-182-5p mimic or miR-NC were co-transfected into CRC cells for 48 h. Cellular luciferase activity was detected using a dual luciferase reporter gene assay kit (E1910, Promega).

### Pull-down assay using biotinylated miRNA

The biotinylated miR-182-5p, miR-151-3p, or control RNA (Guangzhou RiboBio Co., Ltd., Guangzhou, Guangdong, China) was transfected into DLD1 and SW480 cells at a final concentration of 100 nM. Two days after transfection, the whole cell lysates were harvested. Biotinylated RNA complexes were pulled down by incubating cell lysates with streptavidin-coated magnetic beads on a spinner at 4 °C overnight. TRIzol LS Reagent (10,296,010, Gibco) was used to extract RNA from input beads and pull-down beads. The abundance of circ_0008345 in the binding fraction was assessed by RT-qPCR analysis.

### Nuclear and cytoplasmic RNA extraction

Nuclear and cytoplasmic RNA of DLD1 and SW480 cells were extracted according to the instructions of PARISTM Kit (AM1921, Gibco), respectively, followed by reverse transcription and fluorescence real-time quantitative PCR to detect circ_0008345 subcellular localization. β-actin or U6 was used as cytoplasmic and nuclear controls.

### Colony formation and cell proliferation assays

Stably transfected CRC cells were seeded into 6-well plates (200 cells/well). After 14 days of incubation at 37 °C, colonies were fixed using crystal violet (G1063, Beijing Solarbio Life Sciences Co., Ltd., Beijing, China) and counted under a microscope.

Following the instructions in the BeyoClick™ EdU-488 Cell Proliferation Assay Kit (C0071S, Beyotime Biotechnology Co., Ltd., Shanghai, China), transfected CRC cells in 96-well plates (1 × 10^4^ cells/well) were treated successively with the prepared EdU solution and Click reaction solution. The signals were observed under a fluorescent microscope. EdU-positive (EdU^+^) cell rate was analyzed using ImageJ software.

### Cell migration and invasion assays

In migration assays, transfected CRC cells (1 × 10^5^ cells/well) floating in serum-free medium were seeded into the apical chamber of Transwell plates (CLS3513, Corning Glass Works, Corning, N.Y., USA), and the complete medium was added to the basolateral chamber. After 24 h of incubation, the cells were fixed in paraformaldehyde and stained with crystal violet, and then five fields of view were randomly selected under a microscope for observation and counting. In the invasion assay, CRC cells (4 × 10^5^ cells/well) were loaded into the apical chamber pre-coated with Matrigel. Other steps were the same as for the detection of migration.

### Immunoblotting

Protein extraction was performed using RIPA lysis buffer (P0013C, Beyotime) containing protease inhibitors. After quantification using the BCA kit (23,230, Thermo Fisher Scientific Inc., Waltham, MA, USA), proteins (30 μg) were separated using a 10% sodium dodecyl sulfate-polyacrylamide gel electrophoresis and transferred to polyvinylidene difluoride membranes, which were sealed with 5% skim milk powder for 2 h. The membranes were then probed with the primary antibodies against CYP1A2 (1:5000, ab151728, Abcam, Cambridge, UK) and β-actin (1:200, ab115777, Abcam) at 4 °C overnight. The secondary antibody (1:10,000, ab6721, Abcam) was incubated at room temperature for 60 min. Signals were visualized using ECL reagent (WBULS0100, Merck Millipore, Darmstadt, Germany). Relative protein expression was analyzed using ImageJ software.

### Immunohistochemistry

Paraffin-embedded human CRC tissue or paracancerous tissue blocks were cut to 4 μm thickness and stained for immunohistochemistry using the 3,3-diaminobenzidine kit. To eliminate endogenous peroxidase activity, tissue sections were de-paraffinized, rehydrated, and incubated in methanol containing 3% H_2_O_2_ for 15 min at room temperature before performing antigen retrieval at 95 °C for 20 min. The sections were probed with the primary antibody to CYP1A2 (1:250, ab217377, Abcam) at 4 °C overnight. After incubation with secondary antibody (1:1000, ab6721, Abcam) for 0.5 h at room temperature, the slides were incubated with HRP-peroxidase complex for 30 min at room temperature. The reaction products were visualized, and hematoxylin solution (105,174, Merck) was used for counter-staining. Finally, the expression of CYP1A2 protein in the tissues was determined by observing the number of positive cells under the microscope.

### m6A RNA immunoprecipitation (MeRIP)

The Magna MeRIP™ m6A Kit (17-10499, Merck) was used according to the manufacturer’s instructions. Briefly, purified circRNA from the cells was fragmented to ∼ 100 nt using RNA fragmentation reagent and incubated at 94 °C. After fragmentation, the termination buffer was added, followed by standard ethanol precipitation and collection. The m6A antibody (12 μg) was pre-incubated with 50 μL of beads in IP buffer (150 mM NaCl, 0.1% NP-40, 10 mM Tris-HCl, pH = 7.4) for 1 h at room temperature. Next, 6 μg of the RNA fragment was supplemented to the antibody-bead mixture and incubated on a spinner for 4 h at 4 °C. After sufficient washing, the immunoprecipitation mixture was detached using high concentrations of proteinase K. Bound RNA was extracted using the phenol-chloroform method and ethanol precipitation for qPCR analysis. The remaining steps and primer sequences were described in RT-qPCR above.

### RNase R assay

The CRC cell lines were treated with RNase R and then incubated for 30 min at 37 °C. The treated RNA was then reverse transcribed with specific primers (see above) and detected by real-time quantitative PCR assay, as described previously.

### Tumor xenografts in nude mice

All animal experiments were conducted according to the Guide for the Care and Use of Laboratory Animals and were approved by the Ethics Committee of Zhengzhou Central Hospital Affiliated to Zhengzhou University. The report of animal experiments is under the ARRIVE guidelines. BALB/c nude mice were acquired from Vital River (Beijing, China). Transfected DLD1 cell suspension (2 × 10^6^ cells/0.2 mL PBS) was injected into the right abdomen of BALB/c nude mice. The tumor size was recorded every week after injection, and the length (L) and width (W) of the tumors were measured. The tumor volume was calculated using the formula V = (L × W^2^) × 0.5. Four weeks after injection, the mice were euthanized with an intraperitoneal injection of pentobarbital, and the tumors were resected and weighed.

### Statistics

GraphPad Prism version 8.0 (La Jolla, CA, USA) was used for graph drawing and statistical analyses. Paired *t-tests* were conducted for comparisons between any two groups. One-way/two-way ANOVA with Tukey’s post hoc test was performed to compare more than two groups. If not otherwise stated, all values are mean ± SD. All experiments were repeated three times. Differences with *p* < 0.05 were considered significant.

## Results

### Circ_0008345 expression is significantly upregulated in CRC

**Fig. 1:**
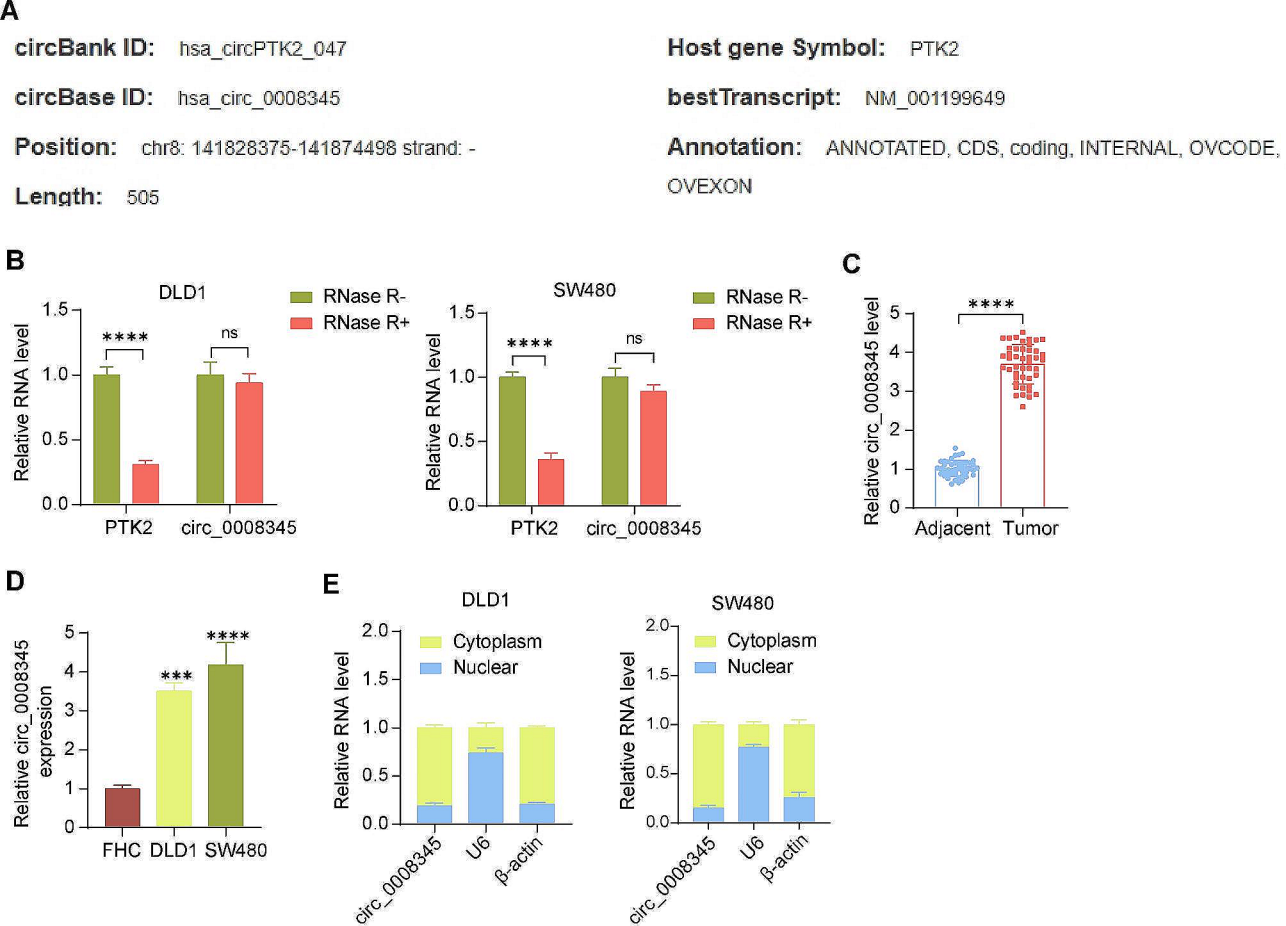
circ_0008345 is overexpressed in CRC tissues and cell lines. **A** PTK2-derived circRNAs were predicted from the circbank database. **B** RNase R treatment of total RNA and analysis of circ_0008345 and PTK2 mRNA resistance levels. **C** The expression of circ_0008345 in CRC and adjacent tissues using RT-qPCR. **D** The circ_0008345 expression in FHC, DLD1, and SW480 cells using RT-qPCR. **E** Subcellular distribution of circ_0008345. Values are the average of three independent experiments. Data are expressed as the mean ± SD. ***p < 0.001, ****p < 0.0001. *p* values were determined by paired t-test (**C**), one-way (**D**), or two-way ANOVA (**B**)

We analyzed PTK2-derived circRNAs using circbank (http://www.circbank.cn/), in which we observed a transcript circ_0008345 (Fig. 1A), whose role in tumors has not been revealed yet. To demonstrate its circular structure, we treated total RNA with RNase R and used linear isoform levels to illustrate the efficacy of RNase R treatment. Circ_0008345 was resistant to RNase R, whereas PTK2 mRNA was not (Fig. 1B). Next, we confirmed that circ_0008345 was higher in CRC tissues compared with normal tissues adjacent to cancer using RT-qPCR (Fig. 1C) and that the expression of circ_0008345 was also higher in DLD1 and SW480 cells compared with FHC cells (Fig. 1D). Further nuclear and cytoplasmic RNA extraction experiments also suggest that circ_0008345 was mainly distributed in the cytoplasm (Fig. 1E) and may be mainly involved in post-transcriptional regulation.

### Circ_0008345 can act as a sponge for miR-182-5p

**Fig. 2:**
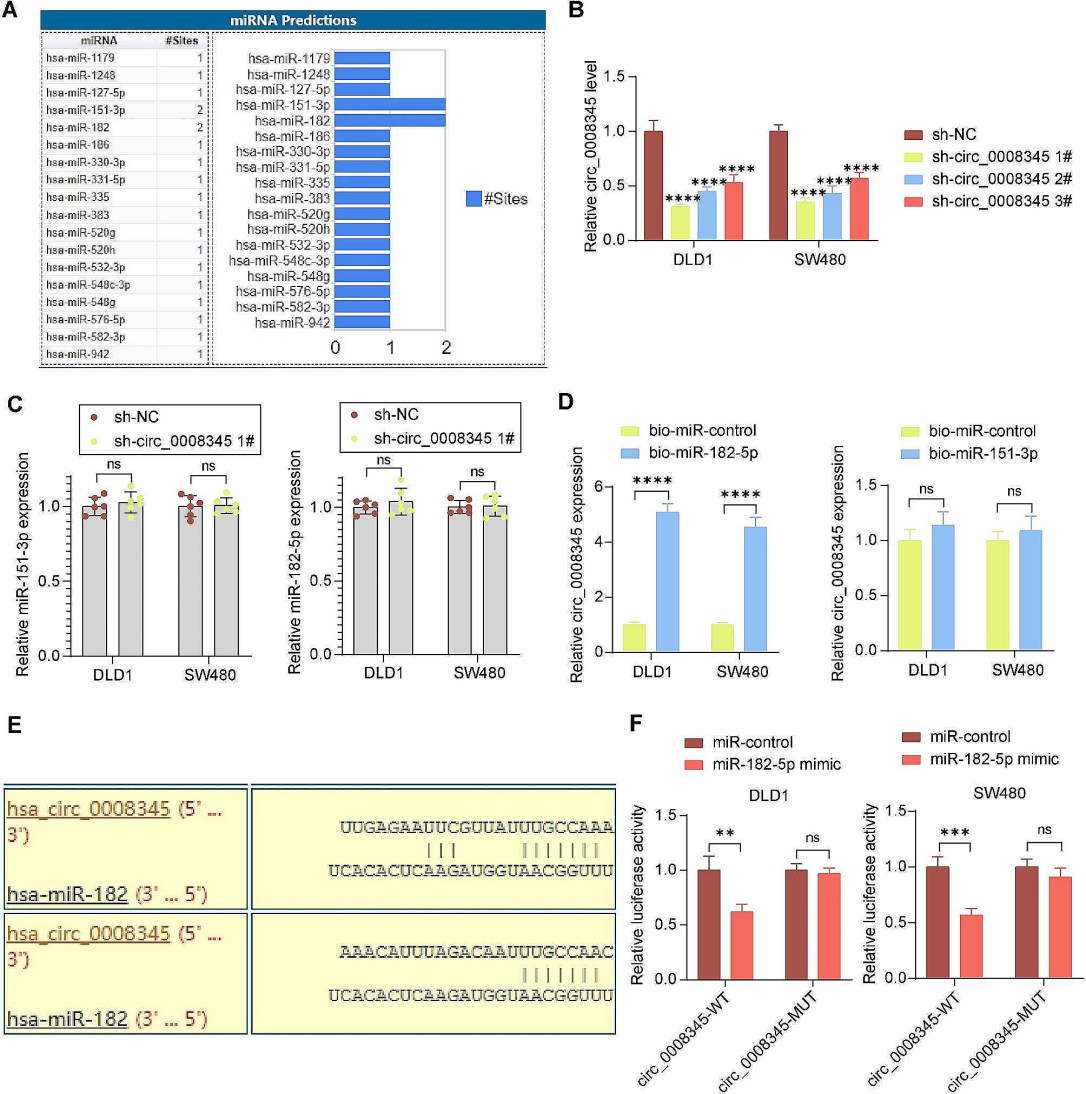
circ_0008345 can sponge miR-182-5p. **A** Search for miRNAs bound to circ_0008345 in circular RNA Interactome. **B** The expression of circ_0008345 in CRC cells treated with three shRNAs targeting circ_0008345 using RT-qPCR (*n* = 3). **C** Effect of knockdown of circ_0008345 on miR-151-3p and miR-182-5p expression detected by RT-qPCR (*n* = 6). **D** The binding relation between biotinylated miR-151-3p or miR-182-5p and circ_0008345 using a pull-down assay (*n* = 3). **E** Two binding sites of circ_0008345 to miR-182-5p in circular RNA Interactome. **F** The binding of miR-182-5p and circ_0008345 using a luciferase reporter assay (*n* = 3). Data are expressed as the mean ± SD. **p < 0.01, ***p < 0.001, ****p < 0.0001. *p* values were determined by two-way ANOVA

The bioinformatics website Circular RNA Interactome (https://circinteractome.irp.nia.nih.gov/index.html) was used to predict the downstream miRNAs of hsa_circ_0008345, and a total of two candidate miRNAs: miR-151-3p and miR-182 (miR-182-5p) were identified since they have two binding sites with hsa_circ_0008345 (Fig. 2A). Three shRNAs of Circ_0008345 were transfected into CRC cells. The RT-qPCR results showed that the knockdown of sh-circ_0008345 1# was the best in both cell lines, so we chose it for the subsequent functional assays (Fig. 2B). Knockdown of circ_0008345 did not significantly affect the expression of the two candidate miRNAs (Fig. 2C). By using a pull-down assay of biotinylated miRNAs, the presence of binding of circ_0008345 to miR-182-5p, but not miR-151-3p, was detected (Fig. 2D). As a competitive endogenous RNA, circRNAs affect miRNA regulation of downstream target mRNAs by competing for miRNA binding sites [18]. Therefore, we chose miR-182-5p, which has a potential binding relationship with circ_0008345, as our study subject. The binding site of circ_0008345 to miR-182-5p was downloaded from the Circular RNA Interactome (Fig. 2E). We then constructed the circ_0008345-WT/MUT vector based on the binding sequence, and the dual-luciferase analysis demonstrated that the miR-182-5p mimic inhibited the luciferase activity of the circ_0008345-WT vector, but not for the circ_0008345-MUT vector (Fig. 2F).

### CYP1A2 is a downstream candidate of miR-182-5p

**Fig. 3:**
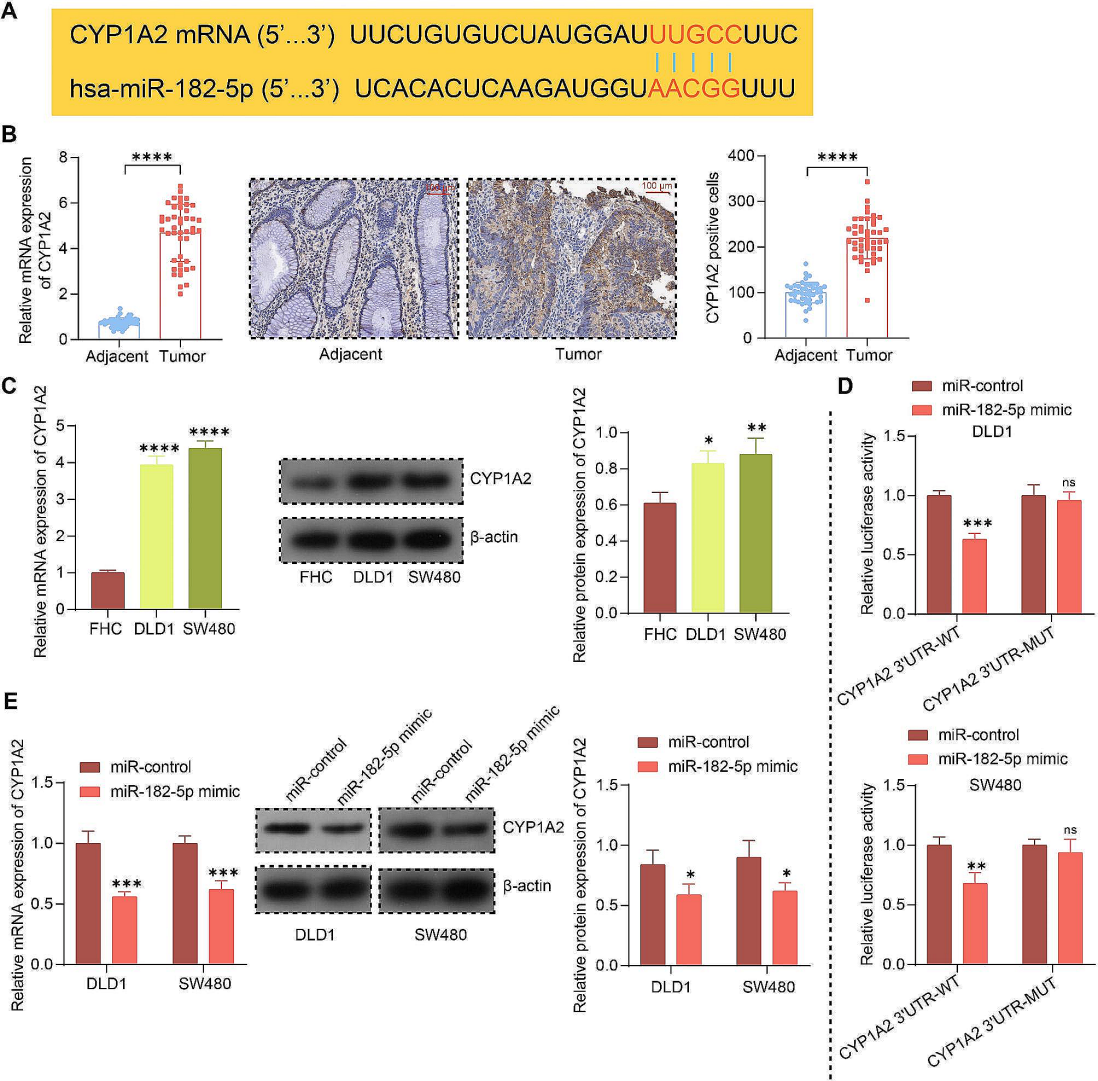
CYP1A2 is a putative downstream target of miR-182-5p. **A** Schematic representation of the complementary sequence between the 3’UTR of CYP1A2 mRNA and miR-182-5p. **B** The expression of CYP1A2 in CRC and adjacent tissues using RT-qPCR and immunohistochemistry. **C** The CYP1A2 expression in FHC, DLD1, and SW480 cells using RT-qPCR and immunoblotting (images of adequate length are shown in the Supplementary File). **D** Verification of miR-182-5p binding to CYP1A2 mRNA by luciferase reporter gene experiments with miR-182-5p mimics and WT/MUT containing the CYP1A2 3’UTR. **E** Effects of miR-182-5p overexpression on CYP1A2 mRNA and protein levels in CRC cells by RT-qPCR and immunoblotting, respectively (images of adequate length are shown in the Supplementary File). Values are the average of three independent experiments. Data are expressed as the mean ± SD. *p < 0.05, **p < 0.01, ***p < 0.001, ****p < 0.0001. *p* values were determined by paired t-test (B), one-way (C), or two-way ANOVA (D and E)

We analyzed the downstream mRNAs of hsa-miR-182-5p in miRWalk (http://mirwalk.umm.uni-heidelberg.de/) and selected the mRNAs with the presence of hsa-miR-182-5p binding site at the 3’UTR for analysis. It was observed that CYP1A2 3’UTR had more binding sites, so CYP1A2 was selected as the downstream target of hsa-miR-182-5p for further analysis. There is a complementary sequence between the 3’UTR of CYP1A2 mRNA and miR-182-5p (Fig. 3A), indicating a possible binding relation. CYP1A2 expression was higher in CRC tissues and cells than in adjacent tissues and FHC cells at both the mRNA and protein levels, respectively (Fig. 3B, C). To confirm that CYP1A2 interacted with miR-182-5p, we constructed luciferase reporter vectors with a WT/MUT binding site for miR-182-5p and CYP1A2 3’UTR. The results showed that only the luciferase activity of the CYP1A2 3’UTR-WT vector could be reduced by the miR-182-5p mimic (Fig. 3D). Next, the results of RT-qPCR and immunoblotting showed that miR-182-5p overexpression significantly reduced CYP1A2 mRNA and protein levels in the cells (Fig. 3E).

### miR-182-5p inhibitor abolishes the anti-tumor effects of sh-circ_0008345 in vitro and in vivo

**Fig. 4:**
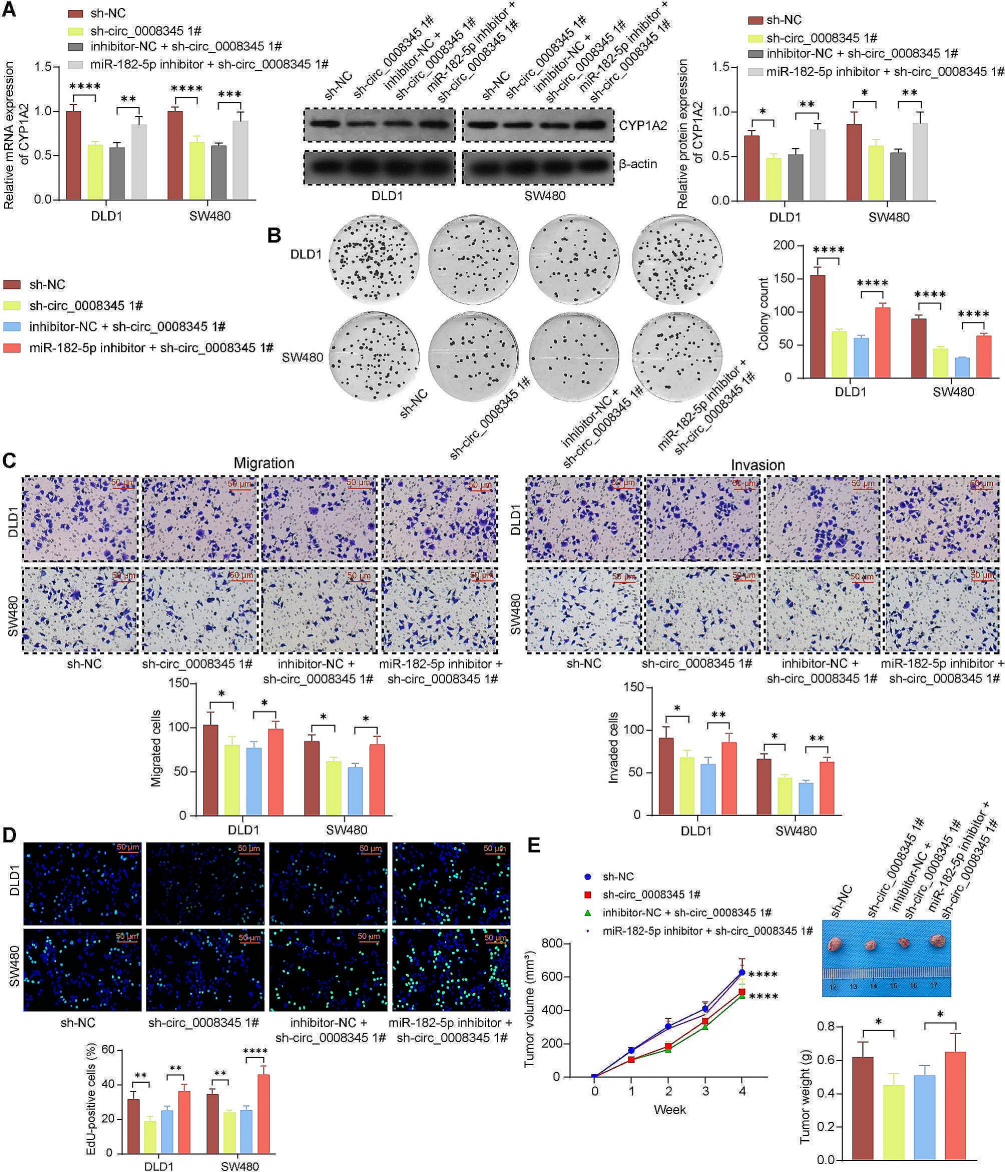
Inhibition of miR-182-5p rescues the impacts of circ_0008345 knockdown on CRC cell malignant behaviors in vitro and in vivo. CRC cells were treated with sh-circ_0008345 alone or in combination with miR-182-5p inhibitor. **A** The mRNA and protein expression of CYP1A2 in response to different infections by RT-qPCR and immunoblotting (images of adequate length are shown in the Supplementary File). **B** The colony formation of CRC cells. **C** The mobility of CRC cells using Transwell assays. **D** The viability of CRC cells using EdU assays. **E** Measurement of tumor volume and weight in mice after subcutaneous injection of transfected DLD1 cells. Values are the average of three independent experiments. Data are expressed as the mean ± SD. *p < 0.05, **p < 0.01, ***p < 0.001, ****p < 0.0001. *p* values were determined by one-way (E) or two-way ANOVA (A-E)

In the presence of sh-circ_0008345, we further treated the CRC cells with miR-182-5p inhibitor to observe the impact on CYP1A2 expression and malignant phenotype of CRC cells. Silencing of circ_0008345 reduced the expression of CYP1A2, which was restored by miR-182-5p inhibitor (Fig. 4A). Moreover, the suppressing effects of sh-circ_0008345 on colony formation were reversed by a miR-182-5p inhibitor (Fig. 4B). As revealed by Transwell assays, sh-circ_0008345 repressed the CRC cells to migrate and invade, while miR-182-5p inhibitor stimulated the migration and invasion in the presence of sh-circ_0008345 (Fig. 4C). In addition, sh-circ_0008345 reduced the CRC cell growth, which was again, overturned by miR-182-5p inhibitor (Fig. 4D, E).

### Depletion of CYP1A2 abrogates the effects of circ_0008345 or miR-182-5p inhibitor in vitro and in vivo

**Fig. 5:**
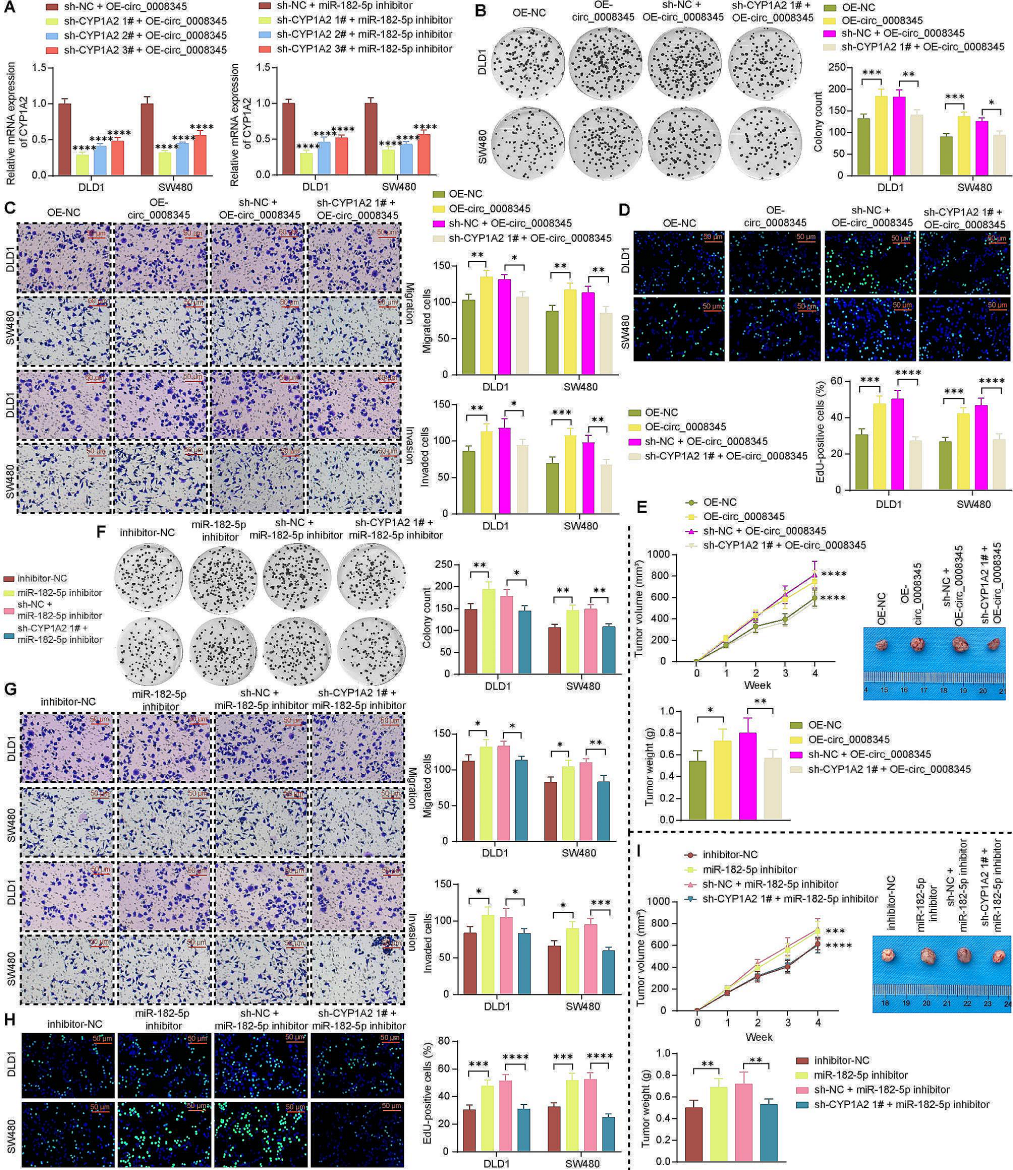
miR-182-5p alters CRC cell growth and motility by binding to CYP1A2. **A** The mRNA expression of CYP1A2 in response to different infections by RT-qPCR. CRC cells were treated with OE-circ_0008345 alone or in combination with sh-CYP1A2. **B** The colony formation of CRC cells. **C** The migration and invasion of CRC cells using Transwell assays. **D** The viability of CRC cells using EdU assays. **E** Measurement of tumor volume and weight in mice after subcutaneous injection of transfected DLD1 cells. CRC cells were treated with miR-182-5p inhibitor alone or in combination with sh-CYP1A2. **F** The colony formation of CRC cells. **G** The migration and invasion of CRC cells using Transwell assays. **H** The viability of CRC cells using EdU assays. **I** Measurement of tumor volume and weight in mice after subcutaneous injection of transfected DLD1 cells. Values are the average of three independent experiments or six mice. Data are expressed as the mean ± SD. *p < 0.05, **p < 0.01, ***p < 0.001, ****p < 0.0001. *p* values were determined by one-way or two-way ANOVA

CRC cells were infected with lentiviral vectors harboring OE-circ_0008345 or miR-182-5p inhibitor + sh-CYP1A2. sh-CYP1A2 1# exhibited the best knockdown effect under both conditions (Fig. 5A), and was chosen for the following assays. Overexpression of circ_0008345 contributed to more colonies formed (Fig. 5B), migrated and invaded cells (Fig. 5C), EdU-positive cells in vitro (Fig. 5D), and larger tumor volume and weight in tumor-bearing mice (Fig. 5E). Consistently, miR-182-5p inhibitor promoted colony formation, cell motility, viability, and enlarged tumor size (Fig. 5F-I). However, the tumor-promoting effects of OE-circ_0008345 or miR-182-5p inhibitor were both aborted by silencing of CYP1A2.

### METTL3 is responsible for the upregulation of circ_0008345 in CRC cells

**Fig. 6:**
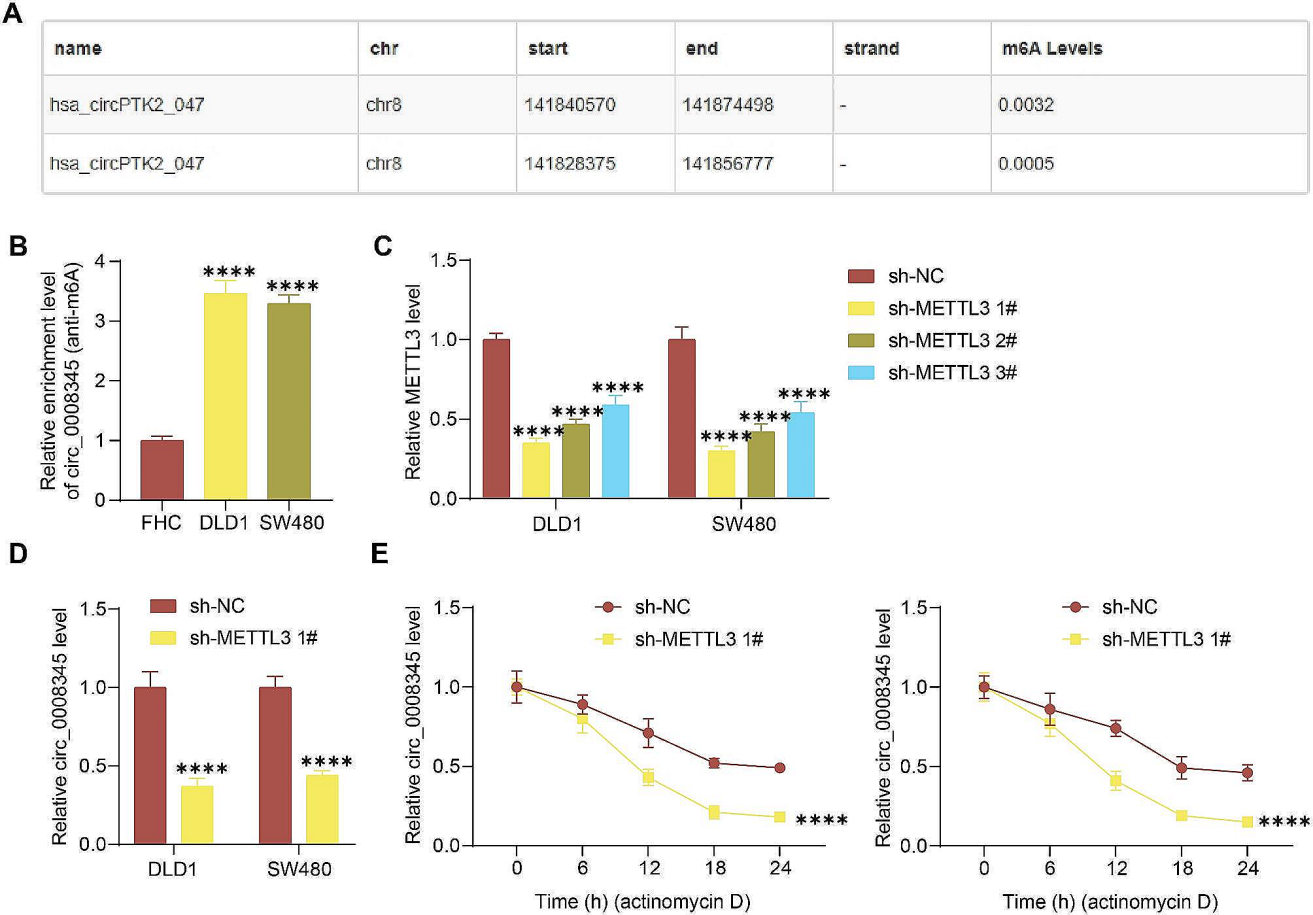
METTL3 mediates circ_0008345 upregulation in CRC cells. **A** The presence of m6A modification site in circ_0008345 predicting using circbank. **B** Detection of m6A-modified RNA levels in cells by MeRIP. **C** The effect of three shRNAs knocking down METTL3 using RT-qPCR. **D** Detection of circ_0008345 expression after knockdown of METTL3 using RT-qPCR. **E** Stability level of circ_0008345 in actinomycin D-treated DLD1 and SW480 cells. Values are the average of three independent experiments. Data are expressed as the mean ± SD. ****p < 0.0001. *p* values were determined by one-way or two-way ANOVA

The possibility of m6A modification of circ_0008345 was observed in circbank (Fig. 6A). Therefore, we first conducted MeRIP. Higher levels of m6A modification in circ_0008345 were observed in CRC cells than in FHC cells (Fig. 6B). The m6A methyltransferase complex consists of multiple “writer” proteins at which METTL3 and METT14 are the core “writers,”, and METTL3 has a catalytic effect, whereas METTL14 does not [19]. METTL3, a m6A methyltransferase, has been reported to cause CRC progression [20, 21]. We therefore hypothesized that METTL3 may regulate circ_0008345 expression in an m6A-dependent way.

We used three lentiviral shRNA vectors to knock down METTL3 in CRC cells, among which sh-METTL3 1# was the most effective (Fig. 6C), and RT-qPCR assay also revealed a significant decrease in circ_0008345 expression after knockdown of METTL3 (Fig. 6D). Then DLD1 and SW480 cells were treated with actinomycin D in the presence of sh-METTL3 1# delivery and it was found that knockdown of METTL3 significantly reduced the stability of circ_0008345 (Fig. 6E).

## Discussion

CircRNAs, owning longer half-lives and stronger strengths than their linear forms, have been identified as molecules that play critical roles in the development of human cancers, including CRC [22, 23]. The current study unveiled the functions of circ_0008345 in CRC for the first time. Moreover, the novel pathway METTL3/circ_0008345/miR-182-5p/CYP1A2 in CRC development was discovered.

As we mentioned above, the role of circPTK2 has been well described in cancer. For example, circPTK2 was overexpressed in most multiple myeloma cell lines compared to normal plasma cells, and circPTK2 upregulation promoted proliferation and migration and inhibited apoptosis in U266 cells [24]. By contrast, circPTK2 knockdown notably repressed cell proliferation and promoted cell apoptosis in gastric carcinoma [25]. Mechanistically, circPTK2 was found to be localized in the cytoplasm of gastric cancer cells and bound to miR-134-5p in gastric cancer cells [26]. Consistently, we observed the cytoplasmic localization of circ_0008345 in CRC cells and later validated the binding relation of circ_0008345 and miR-182-5p. Besides, the anti-tumor effects of sh-circ_0008345 were reversed by miR-182-5p inhibitor in vitro and in vivo. Weiss et al. have recently revealed a positive correlation between miR-182-5p expression and inferior disease-specific survival for patients with oropharyngeal squamous cell carcinomas [27]. miR-182-5p is frequently downregulated in human renal cell carcinoma tissues, and enforced expression of miR-182-5p in renal cell carcinoma cells significantly repressed the proliferation and tumorigenicity [28]. In gastric cancer, miR-182-5p has been reported to be sponged by circ-SFMBT2 to regulate the expression of CREB1 mRNA [29]. In addition, many tumor-related miRNAs were involved in the circRNA-miRNA-mRNA networks in non-cirrhotic hepatocellular carcinoma, including miR-182-5p [30]. More relevantly, miR-182-5p was identified as a downstream molecule of long noncoding AGAP2-AS1 in CRC, thus regulating CRC cell growth and epithelial-mesenchymal transition [31].

MiRNAs are conserved, short (19–22 nt) ncRNAs that combine with Argonaute proteins to form the miRNA-induced silencing complex (miRISC) which uses imperfect base pair complementarity to bind to sequences found mostly in the 3′UTR of target mRNAs and downregulate the expression of that transcript [32]. For instance, Seidl et al. found that miR-182-5p was downregulated in cisplatin-resistant lung adenocarcinoma cells and directly targeted GLI2 [33]. In the present study, CYP1A2 was confirmed as a candidate target of miR-182-5p not only because of their direct binding but also because silencing of CYP1A2 mitigated the oncogenic role of circ_0008345 or miR-182-5p inhibitor. The expression and pharmacological activity of CYP1A2 have been suggested to be post-transcriptionally regulated by has-miR-132-5p in the liver [34]. CYP1A2 encodes a member of the cytochrome P450 superfamily of enzymes, which play an important role in activating and detoxifying many carcinogens and endogenous compounds in the development of CRC [35]. However, its functional role in CRC remains opaque, which partly indicates the novelty of the study.

m6A modification, which is the most prevalent epigenetic methylated modification of ncRNAs, has a profound impact on RNA translation, splicing, and stability [36, 37]. Interestingly, the m6A-mediated circRNA upregulation has been involved in the development of CRC [38, 39]. The effect of m6A RNA modification is controlled by writers, erasers, and readers [40]. m6A writers comprise a multicomponent methyltransferase complex in the nucleus, composed of a core protein heterodimer formed by METTL3 and METTL14, and other regulatory factors to interact with the complex [41]. Moreover, m6A and METTL3 levels were considerably elevated in CRC tissues, and CRC patients with high m6A or METTL3 levels exhibited shorter overall survival [42]. In the present study, we also verified the m6A modification of circ_0008345 predicted in the database was related to METTL3. Besides CRC, METTL3-controlled methylation modification of circRNAs has been implicated in pancreatic ductal adenocarcinoma, hepatocellular carcinoma, and non-small cell lung cancer [43–45], indicating the potential to target METTL3 in cancer treatments. The present study also has certain limitations. First, other writers, including METTL14 [46], METTL16 [47], RBM15 [48], and WTAP [49] have been recently implicated in the progression of CRC. Therefore, further confirmation of the potential effects of these writers on the expression of circ_0008345 will be needed in the future. Moreover, we failed to detect the readers involved in this process, which may also affect circ_0008345 expression.

## Conclusion

**Fig. 7:**
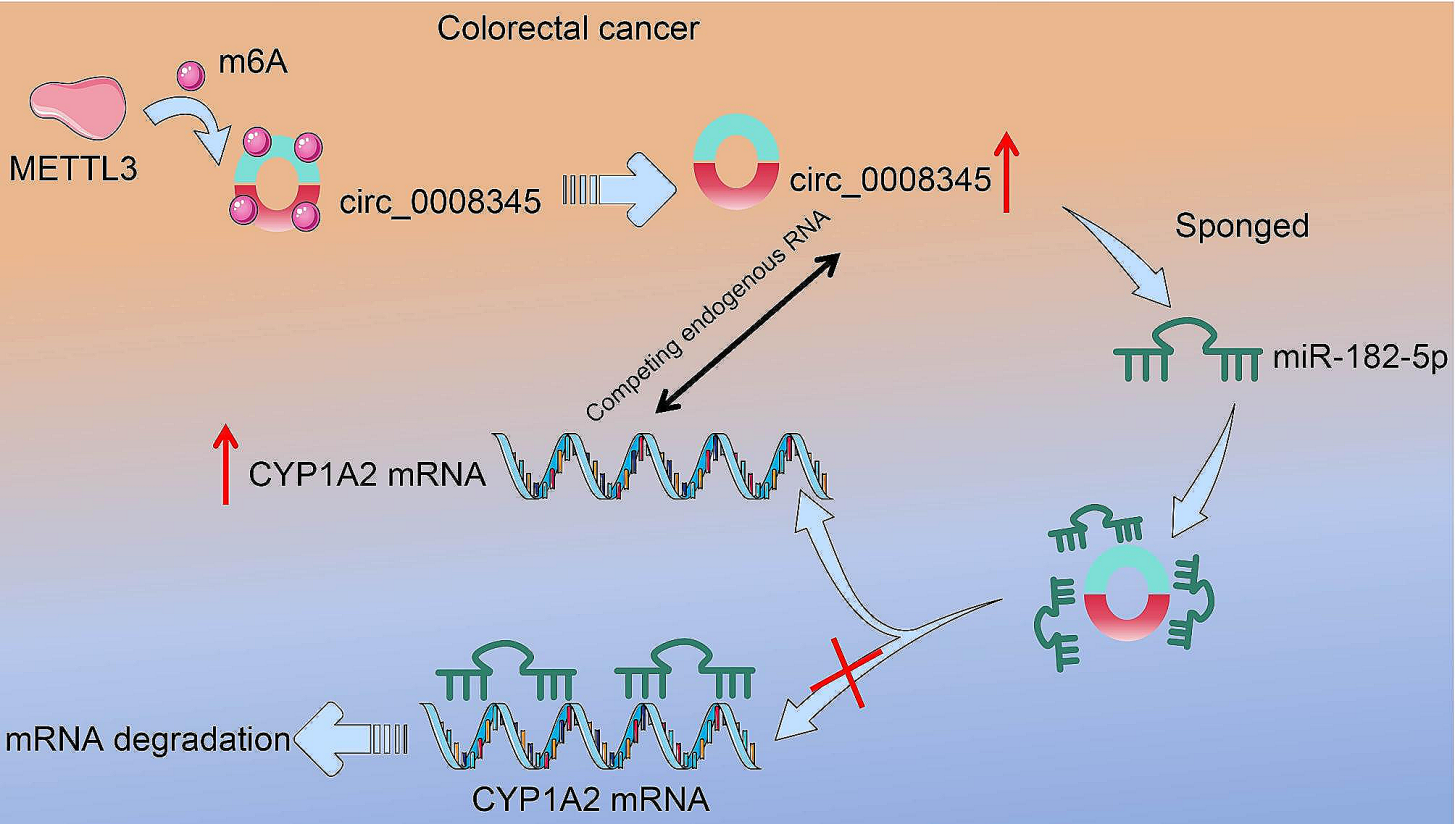
Schematic illustration of circ_0008345 expediting the progression of CRC. METTL3-mediated m6A modification in CRC can upregulate circ_0008345. circ_0008345, as a CYP1A2 ceRNA, can act as a “sponge” and interact with miR-182-5p to increase the mRNA expression of CYP1A2

In summary, our findings suggest that circ_0008345, controlled by METTL3-mediated m6A, promotes the proliferation of CRC through the cross-talk with CYP1A2 mRNA by competing for miR-182-5p (Fig. 7). The clarification of the function of circ_0008345 helps us to understand the mechanisms of CRC progression further.

### Electronic supplementary material

Below is the link to the electronic supplementary material.

Supplementary Material 1

## Data Availability

All datasets used or analyzed during the current study are available from the corresponding author on reasonable request.

## Abbreviations

circRNAs: circular RNA
CRC: Colorectal cancer
miR: microRNA
CYP1A2: Cytochrome P450 1A2
METTL3: Methyltransferase-like 3
MeRIP: m6A RNA immunoprecipitation
