# Supplementary material for: METTL3-induced circ_0008345 contributes to the progression of colorectal cancer via the microRNA-182-5p/CYP1A2 pathway
Source: BMC Cancer. 2024 Jun 14;24:728. doi: 10.1186/s12885-024-12474-5 (PMC11177402; doi:10.1186/s12885-024-12474-5)

Original images of full-length blots

First

Fig 3C: CYP1A2

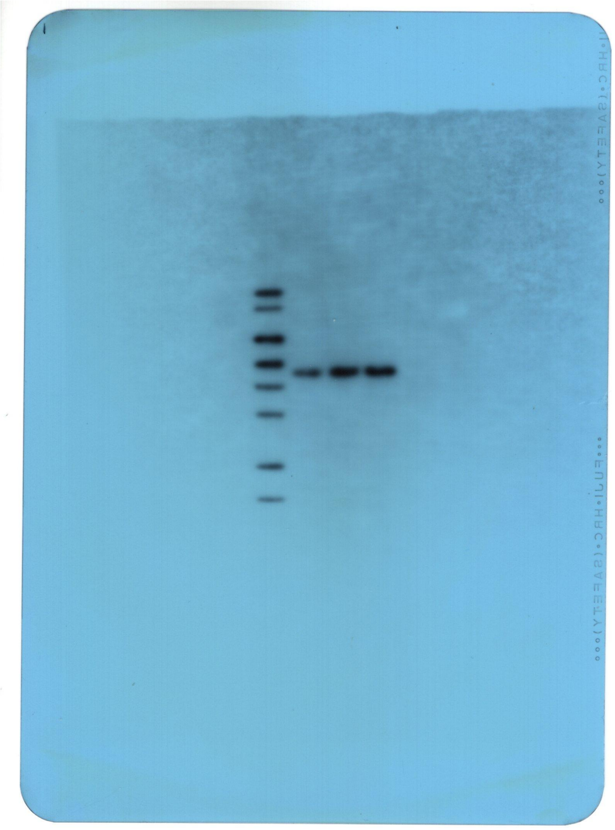

Fig 3C: β-actin

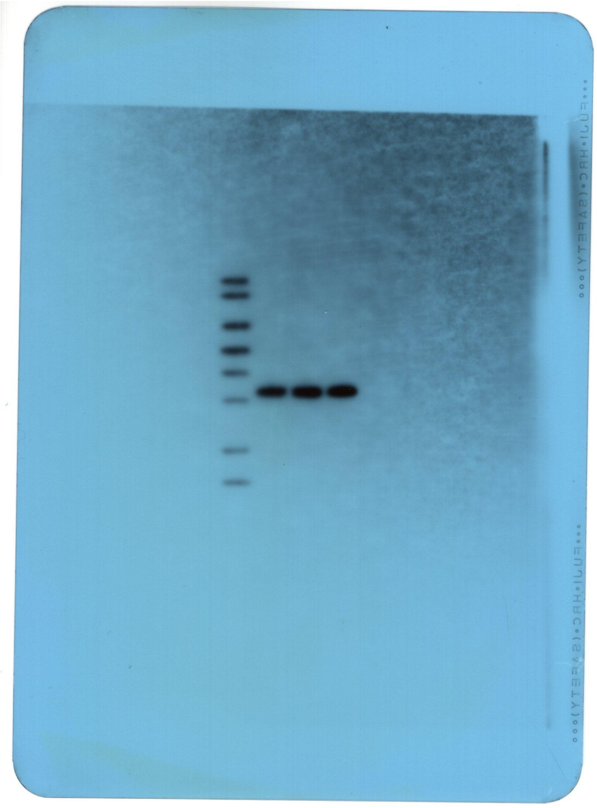

Fig 3E-1: CYP1A2

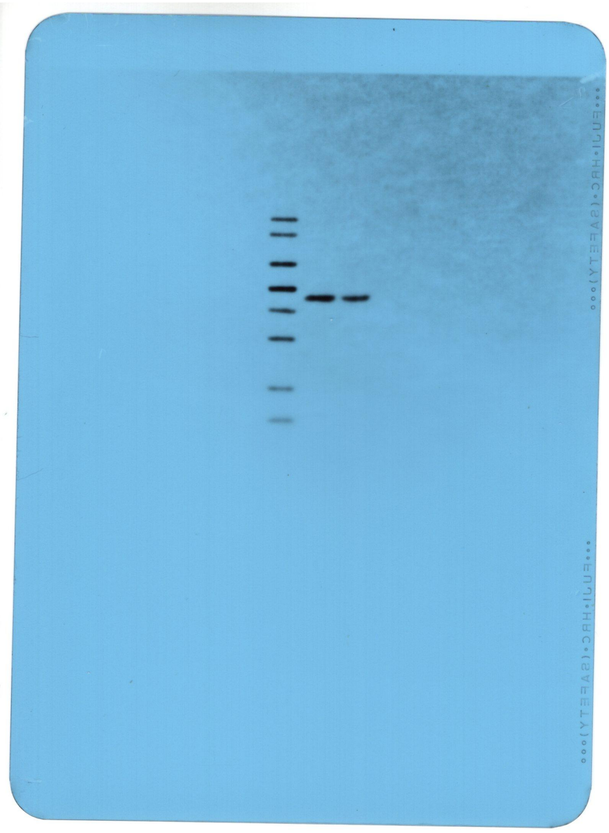

β-actin

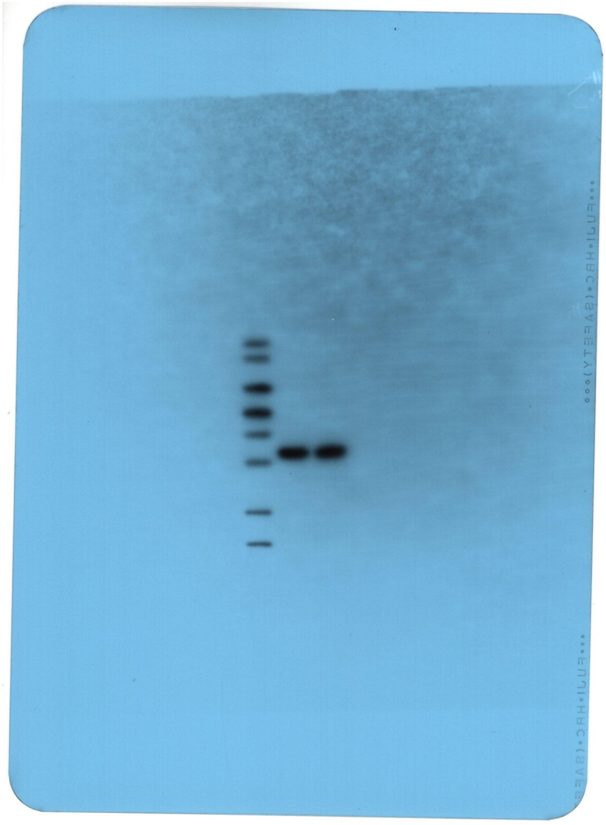

Fig 3E-2: CYP1A2

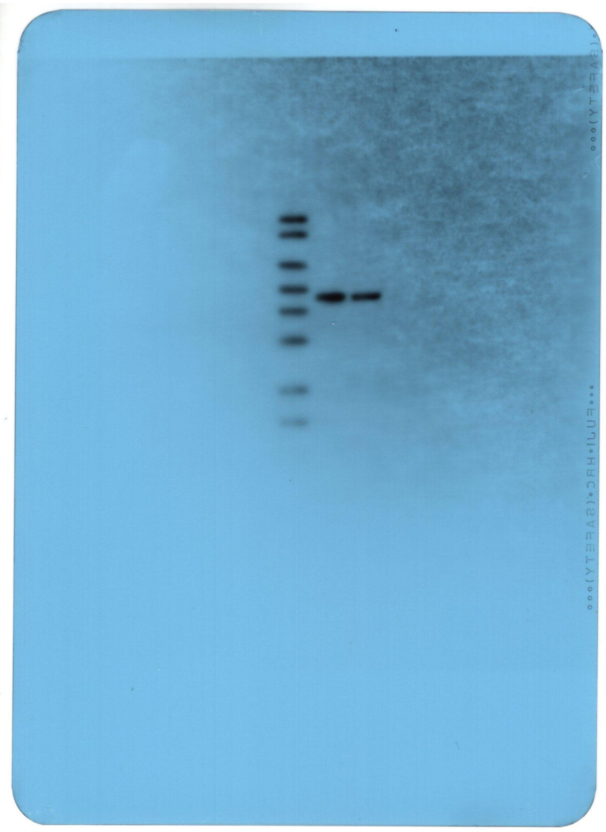

β-actin

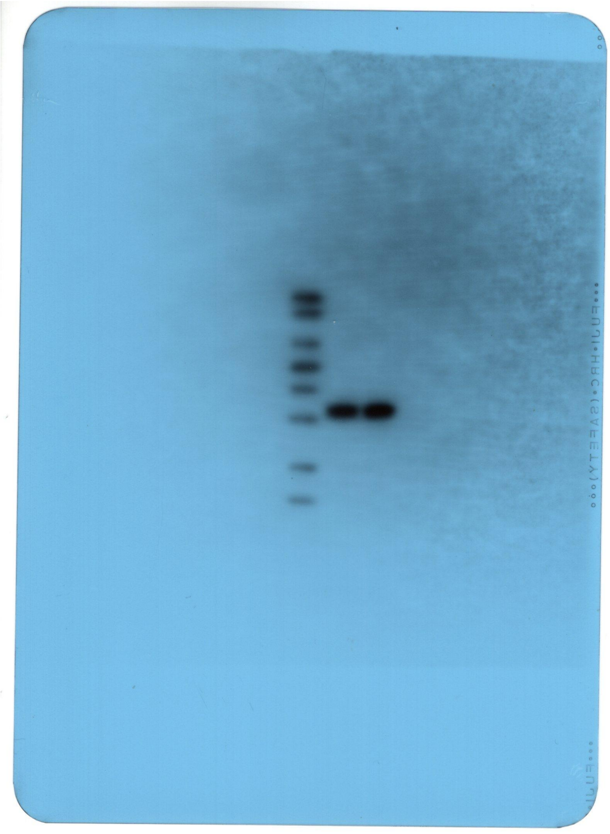

Fig 4A-1: CYP1A2

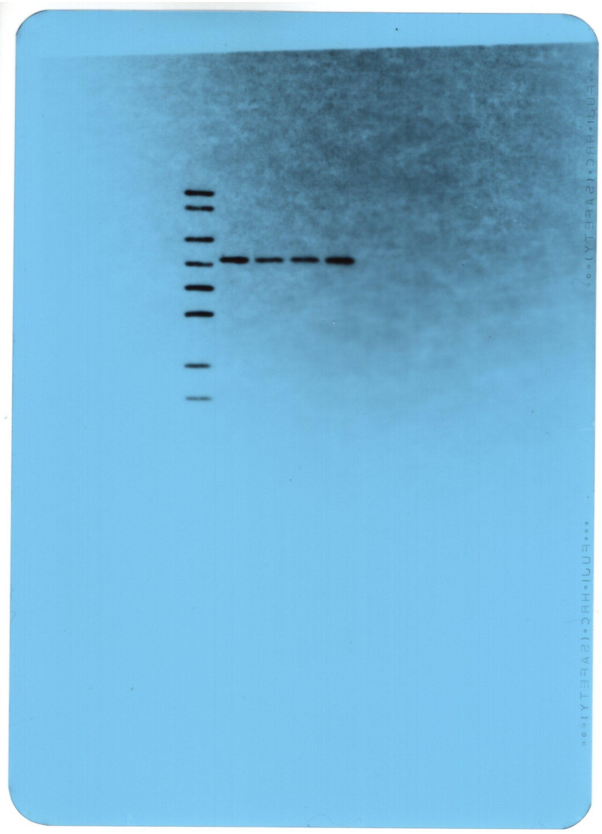

β-actin

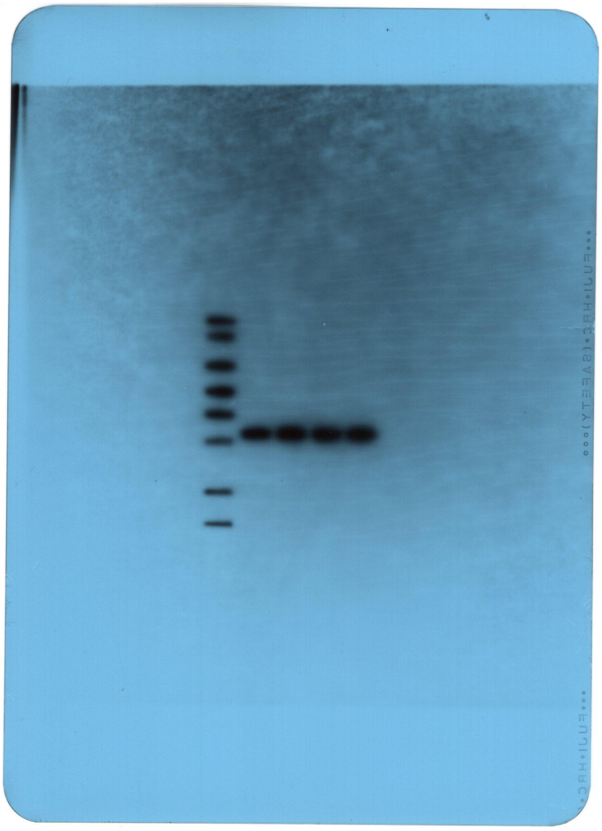

Fig 4A-2: CYP1A2

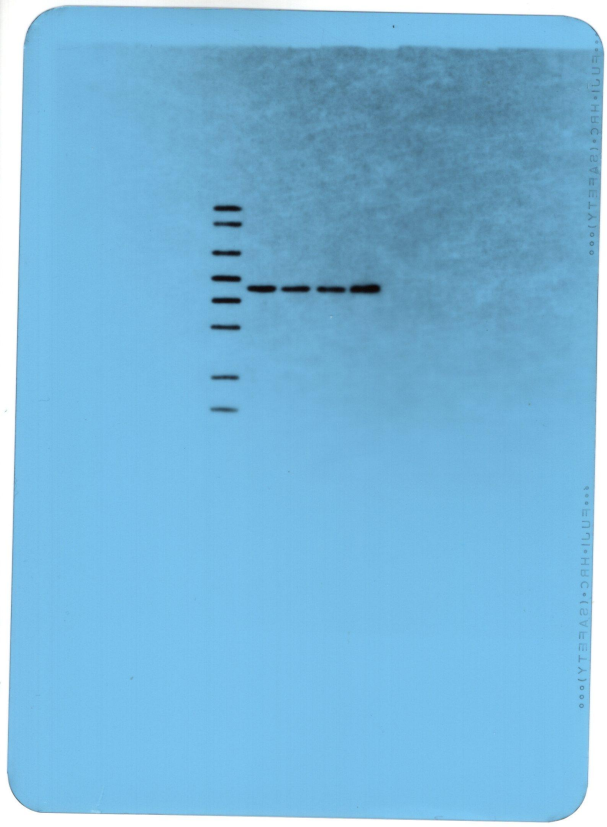

β-actin

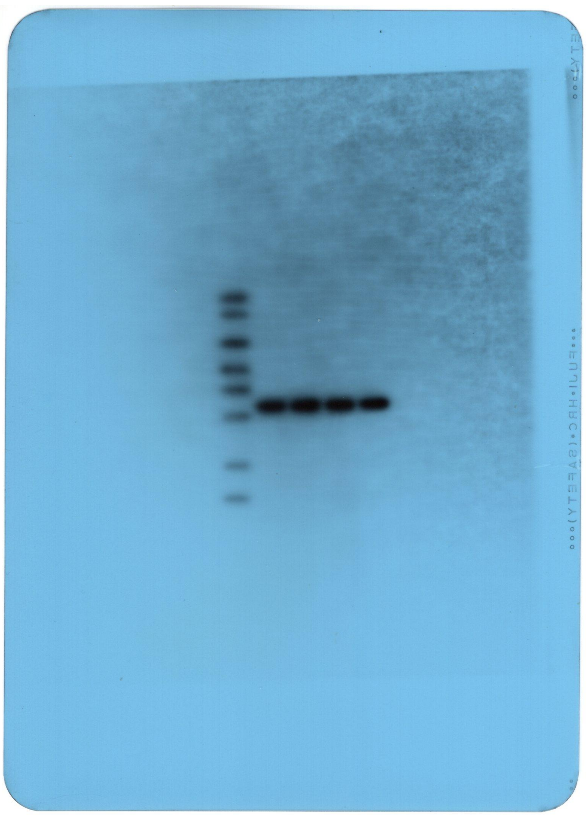

Second

Fig 3C: CYP1A2

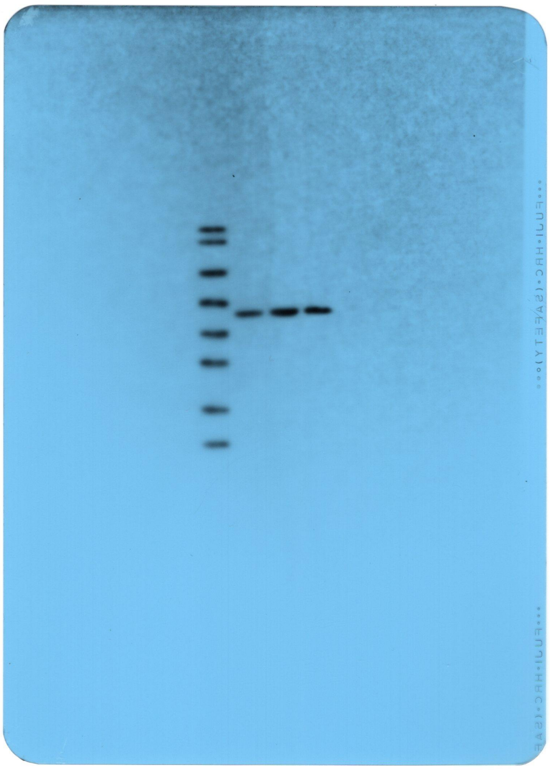

Fig 3C: β-actin

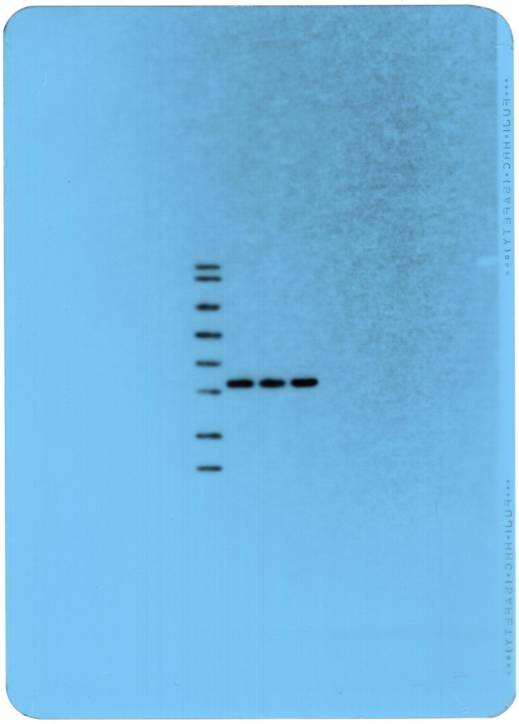

Fig 3E-1: CYP1A2

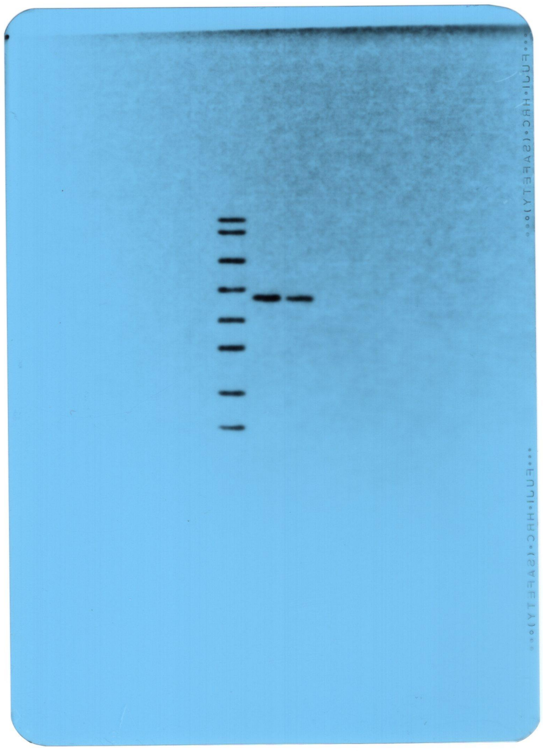

β-actin

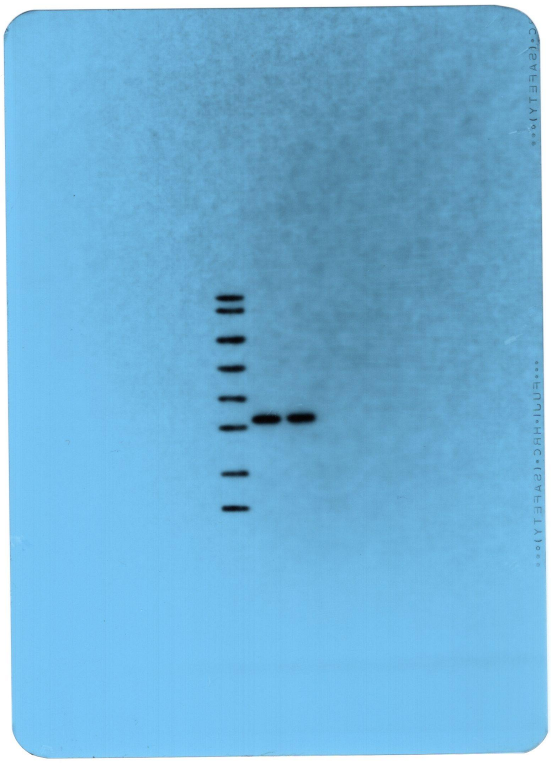

Fig 3E-2: CYP1A2

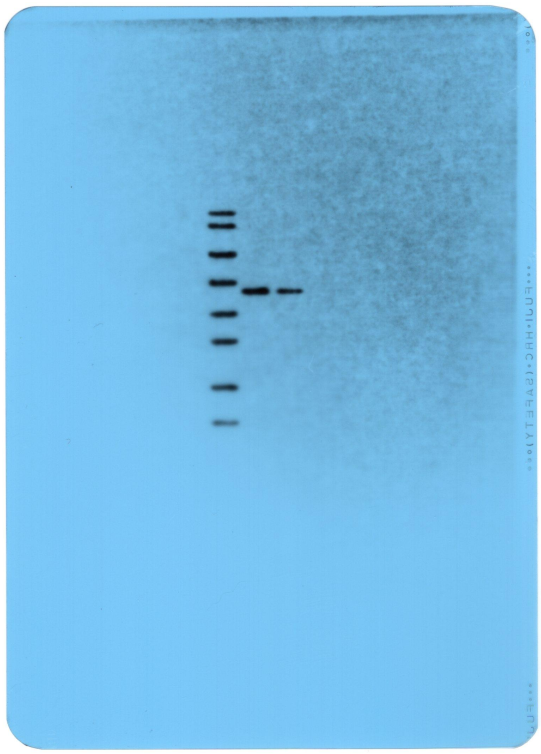

β-actin

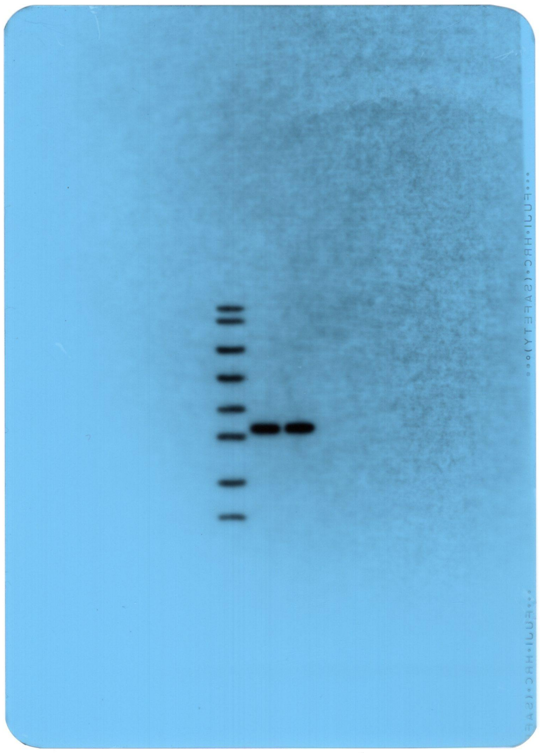

Fig 4A-1: CYP1A2

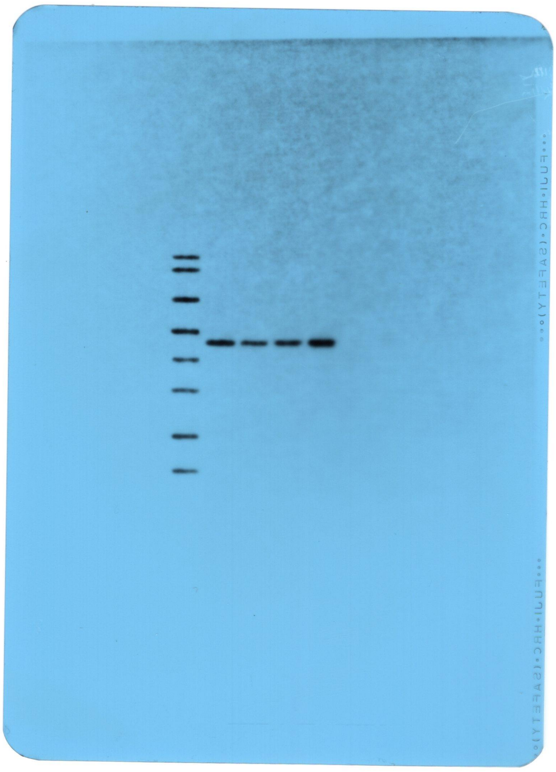

β-actin

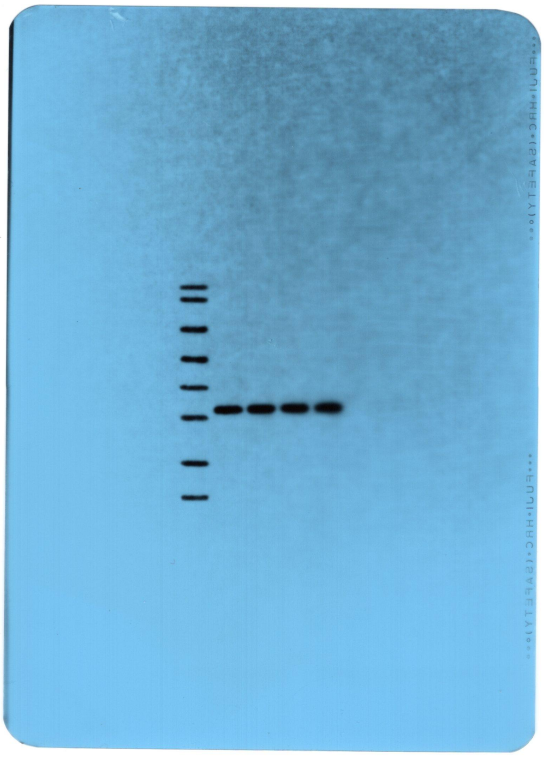

Fig 4A-2: CYP1A2

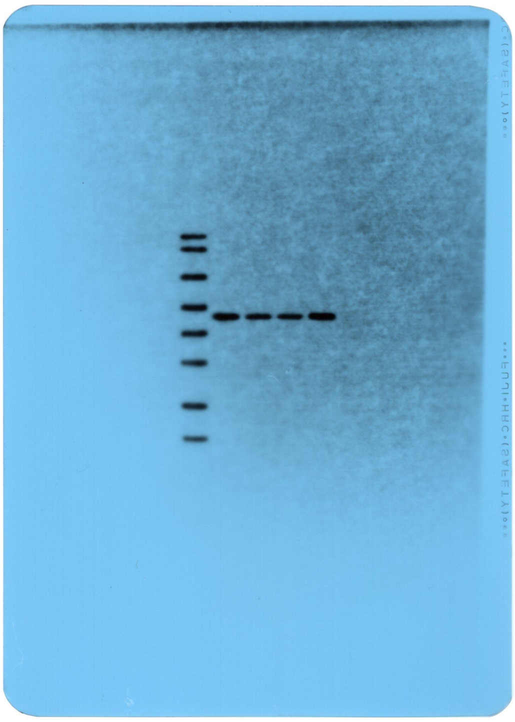

β-actin

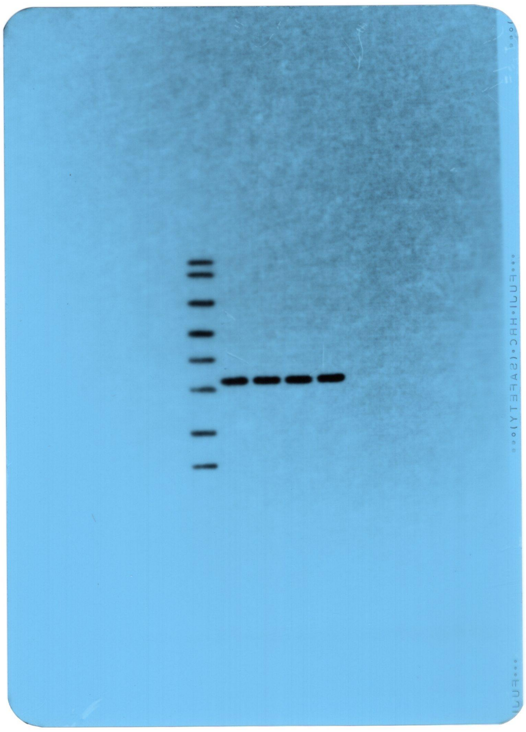

Third
Fig 3C: CYP1A2

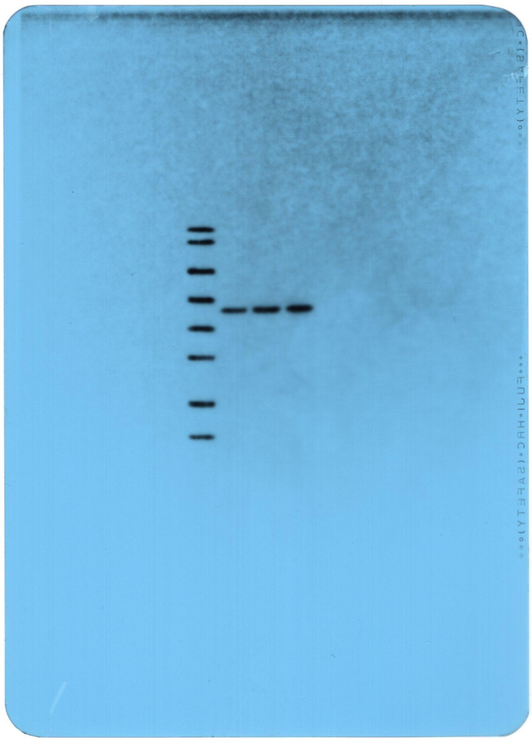

Fig 3C: β-actin

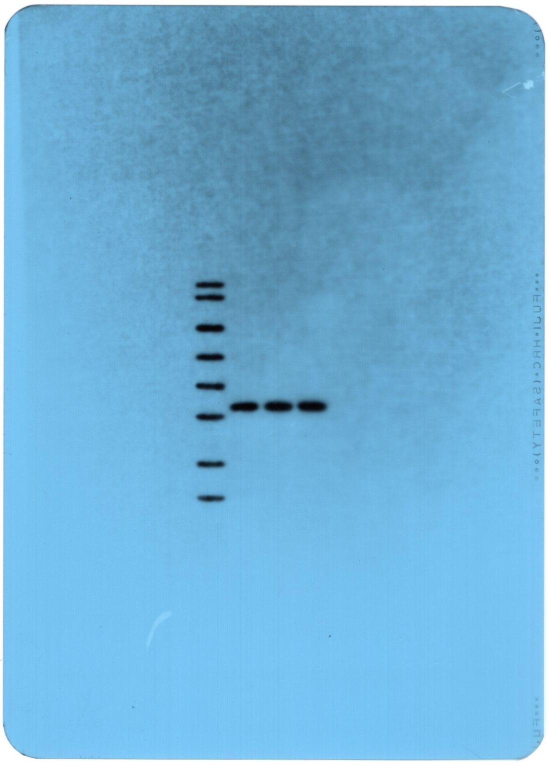

Fig 3E-1: CYP1A2

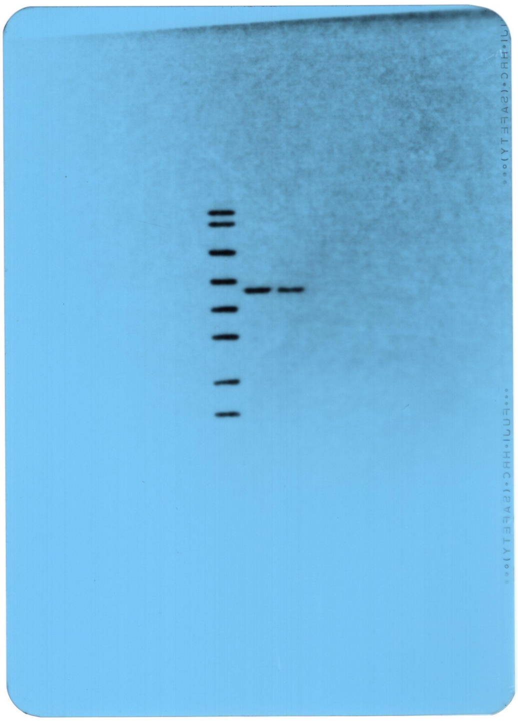

β-actin

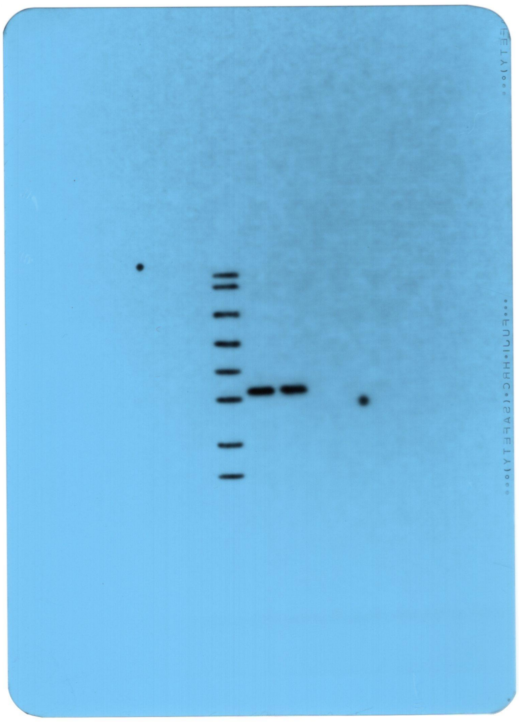

Fig 3E-2: CYP1A2

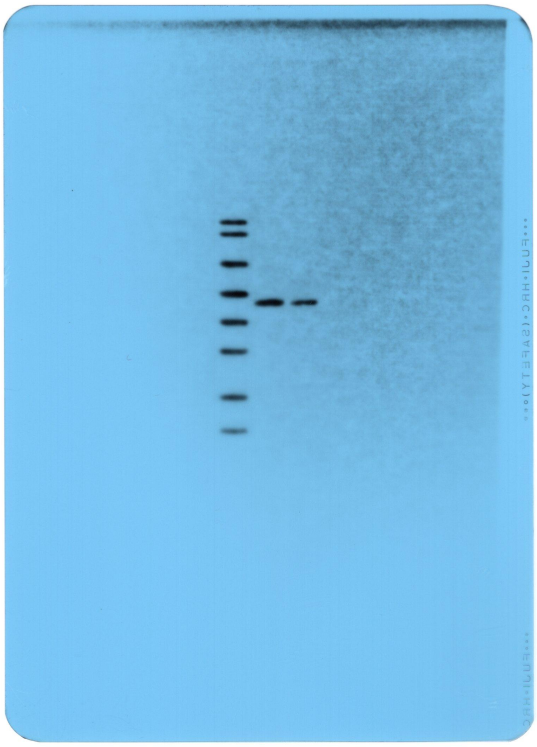

β-actin

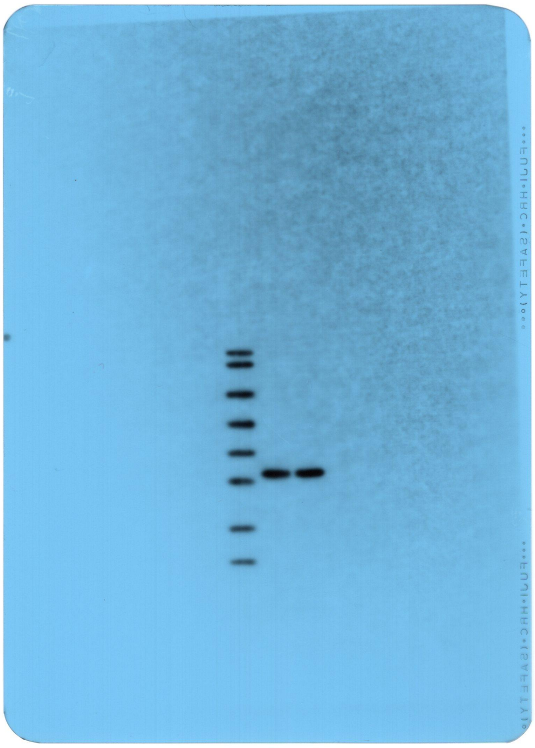

Fig 4A-1: CYP1A2

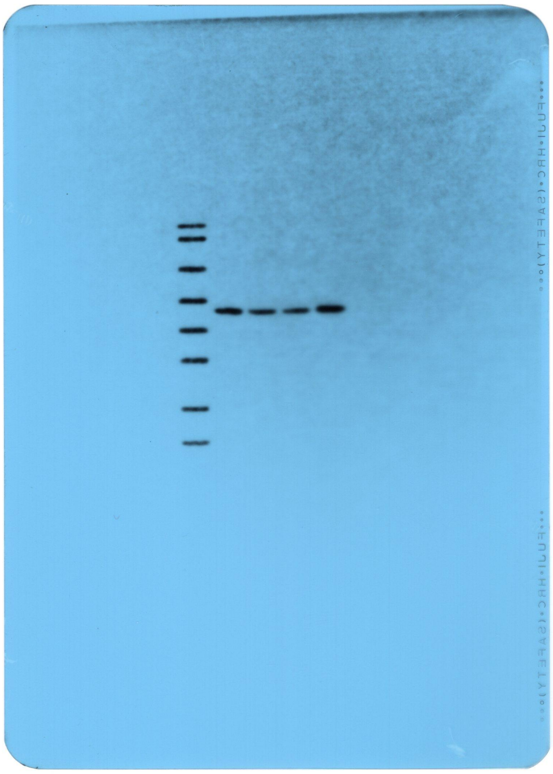

β-actin

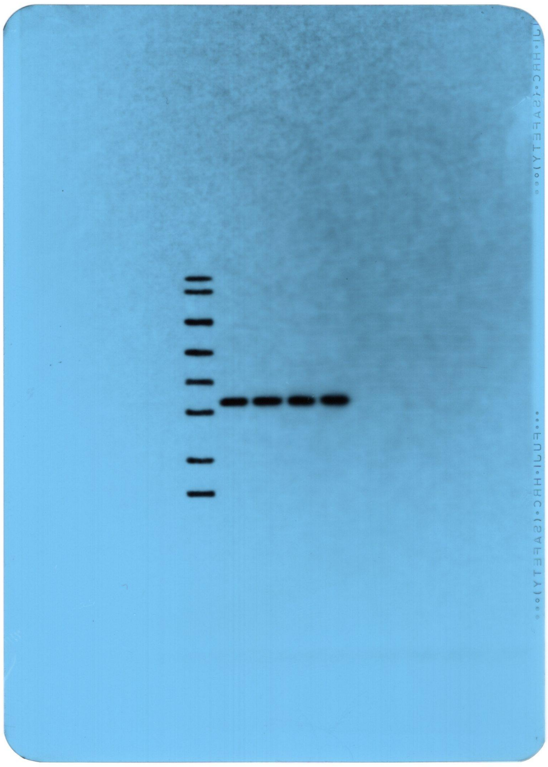

Fig 4A-2: CYP1A2

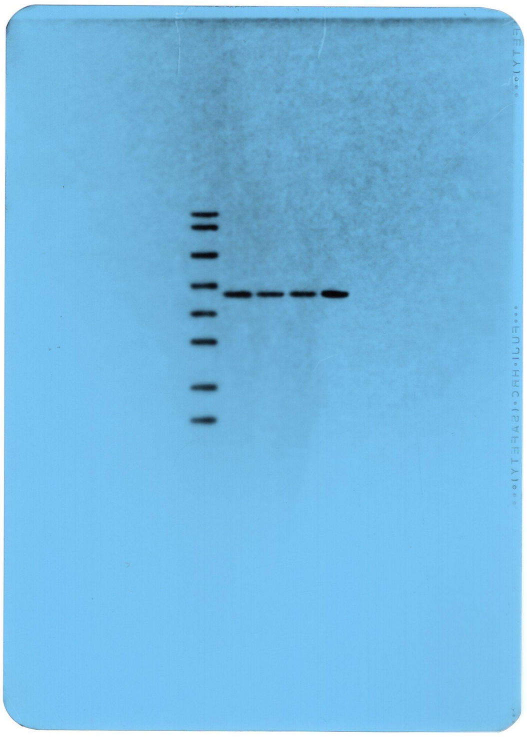

β-actin

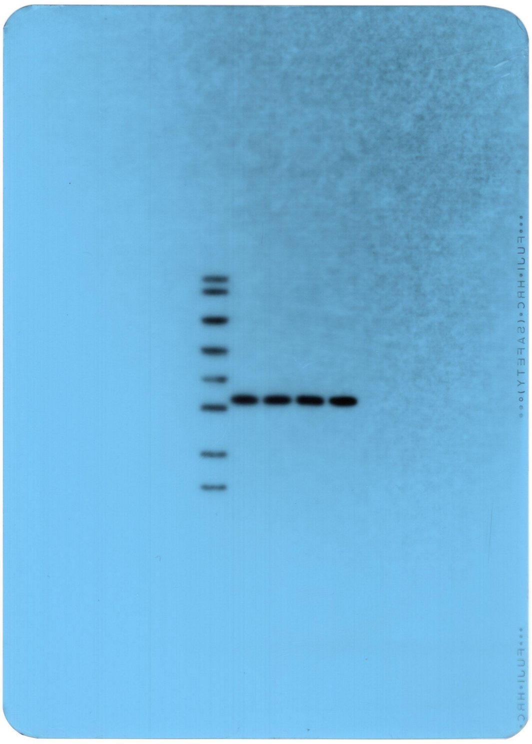

Supplement: Supplementary file 1 — Supplementary Material 1 [file 12885_2024_12474_MOESM1_ESM.pdf]
